# Fetal Cytomegalovirus Associated Intraventricular Hemorrhage: A Rare Prenatal Complication

**DOI:** 10.1055/a-2619-2338

**Published:** 2025-06-11

**Authors:** Vincent D. Tang, Jennifer Heibig, Joanne N. Quiñones Rivera, Albert P. Sarno, Meredith Rochon

**Affiliations:** 1 Division of Maternal-Fetal Medicine, Lehigh Valley Health Network, Allentown, Pennsylvania; 2 Department of Obstetrics and Gynecology, Lehigh Valley Health Network, Allentown, Pennsylvania; 3 Division of Maternal-Fetal Medicine, Department of Obstetrics and Gynecology, Lehigh Valley Health Network, Allentown, Pennsylvania

**Keywords:** cytomegalovirus, intraventricular hemorrhage, fetal ultrasound, prenatal diagnosis, congenital infection

## Abstract

Intraventricular hemorrhage (IVH) is an uncommon manifestation of congenital cytomegalovirus (CMV) infection. We report a case of primary CMV infection associated with preterm labor as well as fetal anemia, thrombocytopenia, and IVH. The finding of unexplained IVH on ultrasound provided an indication for testing of congenital infection. Our case indicates the need for detailed sonographic imaging in pregnancies complicated by preterm labor, as the integration of ultrasound diagnosis can significantly impact management and improve perinatal outcomes.

Cytomegalovirus (CMV) infection is one of the most common antepartum viral infections, complicating 0.2 to 2.2% of all pregnancies. 1 Risk of vertical transmission is estimated to be 35 to 40% and can be associated with significant fetal and neonatal sequelae. 1 Common fetal findings include growth restriction, echogenic bowel, ventriculomegaly, periventricular calcifications, microcephaly, and hydrops. 2 3 Although the risk of vertical transmission after maternal primary CMV infection increases with advancing gestational age, earlier infection increases the risk of symptomatic neonates at birth. 1

Intraventricular hemorrhage (IVH) is a significant cause of neonatal neurologic injury seen in the setting of preterm birth. 4 The pathogenesis of IVH is attributed to the fragility of the germinal matrix and instability of cerebral blood flow seen in prematurity. 5 IVH affects approximately 3 to 25% of infants born prior to 32 weeks. 4 Outside of preterm birth, antenatally diagnosed IVH is very uncommon, with only 240 identified cases in published literature from 1980 to 2019. 5 More specifically, prenatal diagnosis of IVH has rarely been reported in the setting of congenital CMV infection. This report presents a case of fetal CMV-associated IVH, including the antenatal presentation, diagnostic challenges, management strategies, and subsequent outcomes. We aim to enhance the understanding of this rare condition, highlighting the complexities and intricacies associated with precise prenatal diagnosis and management.

## Case Description

Thirty-three-year-old G2P1001 presented with preterm labor and vaginal bleeding at 27 weeks gestation. She reported a low-grade fever for several days prior to presentation with an otherwise negative review of systems. This pregnancy was conceived by in vitro fertilization (IVF) with a donor embryo and assessment of fetal anatomy at 20 weeks did not identify any abnormalities. Her prior pregnancy, also conceived with IVF, was uncomplicated and resulted in a full-term delivery. That child, now 2 years old, was healthy and in daycare. On the initial exam, she was well-appearing with mild discomfort with contractions. A sterile vaginal exam noted the cervix to be 3 cm dilated and 50% effaced with the head at −3 station, and she was noted to have frequent contractions and a reassuring fetal heart rate tracing. She was admitted to the antepartum unit and administered magnesium sulfate for neuroprotection, indomethacin for tocolysis, betamethasone for fetal lung maturity, and ampicillin for group B Streptococcus prophylaxis. She continued to have vaginal bleeding and contractions over the next 24 hours with progression of her cervical dilation to 5 cm after which time her contractions decreased and her cervical exam remained stable. Initial ultrasound showed normal fetal growth, normal fluid, and no evidence of abruption. Heterogeneous echogenic material was seen in the lateral ventricles suspicious for IVH (left greater than right). Echogenic material was also seen in the third ventricle. There was no ventriculomegaly or dilation of the third ventricle, midline shift, mass effect, or parenchymal abnormalities. Middle cerebral artery peak systolic velocity was elevated at 1.99 multiple of the median (MoM) suggesting moderate to severe fetal anemia Initial evaluation included an infectious workup for CMV, parvovirus, toxoplasmosis, herpes simplex virus, and a platelet antibody screen. Due to suspected fetal anemia, plans were made to perform percutaneous umbilical cord blood sampling (PUBS) the following day with intrauterine transfusion (red blood cells and/or platelets) if indicated. However, overnight the patient's contractions increased in frequency and she developed a persistent category II fetal heart rate tracing with recurrent variable decelerations, so the decision was made to proceed with a cesarean delivery. The cesarean delivery was uncomplicated, and she delivered a liveborn male weighing 1,235 g with 1 and 5 minutes APGAR scores of 2 and 8, respectively. Cord blood analysis confirmed fetal anemia (hemoglobin: 9.1 g/dL) and thrombocytopenia (platelets: 67,000/cmm). Significant leukocytosis was also noted (WBC: 63,000/cmm). The neonate required critical care admission due to prematurity with acute respiratory distress requiring intubation and ventilation; transfusion of red cells and platelets was also performed. Approximately 8 hours after delivery maternal serology results returned consistent with an acute primary CMV infection. Additional findings consistent with CMV infection included positive placental CMV immunostain and neonatal saliva CMV polymerase chain reaction (PCR) positive. The neonate was treated with valganciclovir 16 mg/kg twice daily and therapy is planned for 6 months. Initial neonatal head ultrasound on the day of delivery found nonacute bilateral grade 3 germinal matrix hemorrhages and mild ventriculomegaly (left more than right) with concurrent chemical epididymitis. Subsequent exams over the next 9 weeks showed the development of posthemorrhagic hydrocephalus which later returned to mild ventriculomegaly, no evidence of additional bleeding, and no parenchymal abnormalities. The initial hearing screen was negative. Newborn was eventually discharged in stable condition and followed closely as an outpatient.

## Discussion

While IVH is more commonly diagnosed postnatally following a traumatic or preterm birth, this case of congenital CMV represents a rare complication of IVH identified antenatally. The incidence of fetal IVH is estimated to occur between 1 and 5 per 10,000 pregnancies. 6 The paucity of existing literature regarding germinal matrix or IVH is limited to descriptive or observational studies. As a result, very little is understood regarding risk factors when IVH occurs antepartum. Reported associations of fetal IVH include pregnancies affected by multifetal gestation, fetal growth restriction, congenital anomalies, maternal infection, hypertensive disorders, and abnormal amniotic fluid volume. 6 Causative mechanisms of IVH are hypothesized to result from altered fetal hemodynamics, clotting, or cerebral autoregulation. 6 Severe complications such as twin-to-twin transfusion syndrome and neonatal alloimmune thrombocytopenia (NAIT) are conditions that are better understood to be risk factors for fetal neurologic injury and intracranial bleeding, respectively. 6

**Fig. 1:**
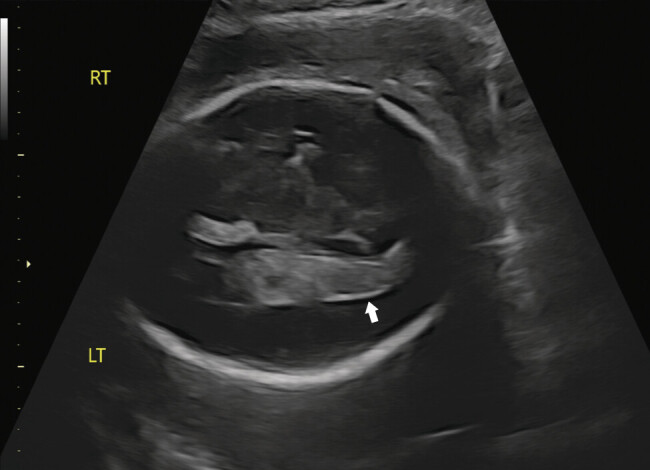
Fetal head ultrasound in grayscale of left lateral ventricle demonstrating heterogeneous and echogenic portions of choroid plexus suspicious for intraventricular hemorrhage (white arrow). LT, left; RT, right.

Ultrasound diagnosis of IVH can be a challenging endeavor. Acute hemorrhage appears as an echogenic and homogenous lesion. With time, the appearance becomes less echogenic but more heterogenous. Additionally, IVH can be associated with ventriculomegaly or periventricular calcifications from obstruction of cerebrospinal fluid and intracranial vasculature. Identification of intracranial lesions after a normal mid-gestation anatomy ultrasound requires additional or subsequent antenatal imaging. In the setting of pregnancy complications or co-morbidities, indicated ultrasound surveillance allows for additional opportunities for detecting IVH. ( Fig. 1 )

**Fig. 2:**
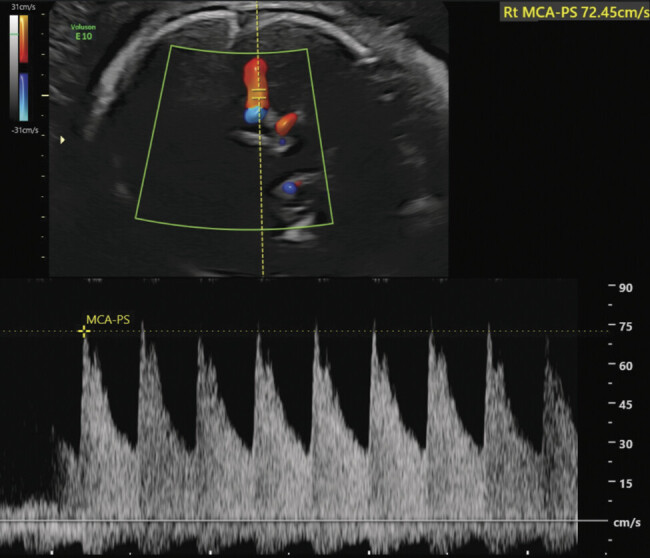
Fetal middle cerebral artery peak systolic velocity by pulse wave color Doppler ultrasound. Peak systolic velocity is 72.45 cm/second, correlating with 1.99 MoM, indicating risk for fetal anemia.

In this case report, the integration of diagnostic ultrasound as a part of the patient's clinical workup helped identify fetal IVH in the setting of her preterm labor. The ultrasound finding of IVH prompted the utilization of middle cerebral artery Doppler to assess the risk of fetal anemia. Middle cerebral artery peak systolic velocity greater than 1.5 MoM suggests moderate to severe fetal anemia. These sonographic markers led to a broad clinical differential, which included the possibility of congenital infection, NAIT, other genetic thrombocytopenia, germinal matrix hemorrhage, and abnormal vasculature. ( Fig. 2 )

Prenatal diagnosis of congenital CMV infection solely by ultrasound imaging alone is generally not able to consistently diagnose intrauterine infection. 7 However, the presence of intracranial and/or multiple abnormalities such as growth restriction, echogenic bowel, microcephaly, hepatosplenomegaly, hepatic calcifications, and hydrops can be more supportive. 7 When CMV infection is suspected, serology testing with CMV-specific IgG, IgM, and IgG avidity should be offered. 8 Diagnosis of maternal primary CMV infection should be on the basis of the detection of both CMV IgM and low-to-moderate avidity of CMV IgG antibodies. 8 Our patient reported a several-day history of low-grade fever with an otherwise negative review of systems. Primary infection during pregnancy is asymptomatic in approximately 75 to 95% of women. 9 When symptomatic, CMV infection may involve mild febrile illness with associated fatigue, myalgia, headache, or rhinitis. 9

Prenatal diagnosis of fetal CMV infection can be made by nucleic acid test assays such as PCR after 20 weeks gestation, once fetal urination is well established, and at least 6 to 8 weeks from the time of maternal CMV infection. 10 In congenital infection, the sensitivity of CMV PCR from fetal urine excreted into the amniotic fluid is roughly 70%. 10 Amniocentesis for CMV is recommended when there is maternal CMV infection during pregnancy or when there are abnormalities on ultrasound consistent with fetal infection. Postnatal diagnosis of congenital CMV in affected neonates should include PCR of saliva or urine within the first 21 days of life, with saliva as the preferred method. 10 In this case report, CMV testing by maternal serology and newborn saliva PCR were consistent with primary maternal infection that resulted in congenital infection. Intended amniocentesis with PUBS was planned but unable to be completed due to indicated cesarean delivery.

Recent literature has reported the utilization of antenatal cord blood testing by PUBS or cordocentesis to supplement fetal assessment in CMV congenital infection. In one study, prenatal diagnosis of congenital CMV by fetal blood had a similar sensitivity of 75.6% compared with 72.7% by amniotic fluid. 7 However, due to the higher complication rate associated with PUBS sampling, amniocentesis is still considered the method of choice for diagnosis of fetal CMV infection. In addition to virologic testing, fetal blood can provide other laboratory information such as platelet count, β-2 microglobulin, and liver enzymes. 7 Fetuses affected by CMV can present with thrombocytopenia, elevated β-2 microglobulin, and transaminitis compared with noninfected fetuses. 7 Reported thresholds such as β-2 microglobulin >14 mg/L or platelet counts <120,000/cmm were associated with severe ultrasound abnormalities. 7 A separate recursive analysis completed on a case-control study described that the presence of moderate thrombocytopenia <120,000/cmm, CMV DNA load >5 log10 IU/mL, or β-2 microglobulin >12 mg/L in cases with severe brain abnormalities on prenatal imaging achieved a positive predictive value (PPV) of 100%. 11 Even in cases without severe intracranial abnormalities at prenatal imaging, moderate thrombocytopenia had a PPV of 83% for symptoms at birth. 11 Conversely, the contribution of PUBS could be particularly useful for fetuses without any ultrasound or laboratory abnormalities as the negative predictive value is close to 100% for moderate to severe symptomatic infections. 11 Although these studies are observational, the results allude to the possible prognostic value of antenatal imaging and PUBS as helpful supplemental tools for identifying fetuses at increased risk for severe disease. Information obtained antenatally can change ongoing fetal surveillance and is informative for the neonatal management of the newborn.

**Fig. 3:**
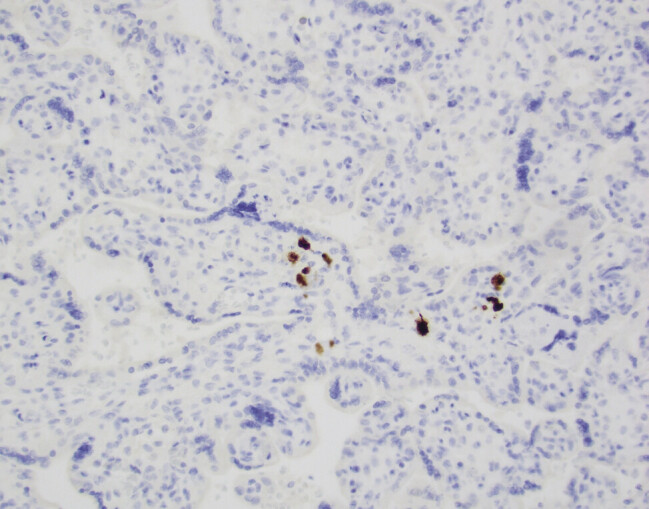
Positive immunoperoxidase stain for CMV on placental pathology (positive stain colored brown).

Pathology examination of the placenta also demonstrated CMV-positive cells by immunoperoxidase stain. Congenital infection occurs through placental-mediated vertical transmission. 1 As the first organ exposed to the CMV virus, the placenta initially serves as a barrier before succumbing as a reservoir for viral replication. 1 The CMV virus crosses the placenta and replicates in the tubular epithelium of the kidneys, with a trophism for the reticuloendothelial and central nervous system. 1 CMV-infected placentas demonstrate histopathological changes that affect cellular morphology. The classic histopathologic finding associated with СMV infection is villitis, but may also include plasma cell deciduitis, sclerosis of the villous capillaries, chorionic vessel thromboses, necrotizing villitis and hemosiderin deposition in the villous stroma. 12 13 As demonstrated by the obstetric outcome in this case report, congenital CMV infection with placental pathology is associated with an increased risk for preterm delivery. 12 ( Fig. 3 )

While the relationship between infection severity and newborn outcomes of antenatally diagnosed IVH is not fully understood, maternal CMV infection in the first trimester is well established as a risk factor for the most severe fetal symptoms. 1 6 An estimated 19 to 29% of fetuses infected in the first trimester are likely to have neurologic lesions, with an increased risk of late-onset sequelae such as cognitive, motor, and sensory impairments. 11 Infection in the second trimester may also lead to symptoms in the fetus or the newborn. 1 Following delivery, the newborn was admitted to the neonatal intensive care unit due to complications of prematurity and CMV infection. Initial head ultrasound demonstrated grade 3 IVH, meaning the hemorrhage occupied >50% of the lateral ventricle area with acute ventricular dilation. The infant's posthemorrhagic hydrocephalus improved to mild ventriculomegaly without any evidence of parenchymal abnormalities prior to discharge home. He was treated with valganciclovir for 6 months of total therapy. Long-term sequelae of congenital CMV infection in symptomatic neonates can include sensorineural hearing loss and neurodevelopmental delay. 9 His initial hospital and subsequent outpatient hearing evaluations were normal. He was seen at 9 months of age (corrected age 6 months) at our institution's newborn specialty center for outpatient surveillance. Bayley Scales of Infant and Toddler Development (BSID) was utilized for developmental assessment. BSID provides a comprehensive evaluation of an infant's motor, speech, and cognitive milestones. 14 For his adjusted age, our infant was meeting his cognitive and motor milestones but demonstrated delays in his speech and expressive language. Plan of care recommendations involved referral to speech therapy through the Early Intervention program.

IVH is an uncommon manifestation of congenital CMV infection. In this case, our patient in preterm labor had imaging suggesting IVH and fetal anemia which was subsequently diagnosed with primary CMV infection. The inflammatory process of the viral infection likely led to the development of preterm labor. The IVH may have occurred due to CMV-associated thrombocytopenia; alternatively, the IVH may have been the primary event (perhaps due to inflammation) leading to anemia and thrombocytopenia. Suspicion for congenital CMV infection, especially in the cases of asymptomatic mothers relies on fetal ultrasonographic findings to initiate workup. Our case indicates the need for detailed sonographic imaging in pregnancies complicated by preterm labor, as ultrasound findings can significantly impact management.
